# Retraction: Synthesis, Characterization and In Vitro Study of Biocompatible Cinnamaldehyde Functionalized Magnetite Nanoparticles (CPGF Nps) For Hyperthermia and Drug Delivery Applications in Breast Cancer

**DOI:** 10.1371/journal.pone.0280492

**Published:** 2023-01-10

**Authors:** The PLOS ONE Editors

Following the publication of this article [1], concerns were raised regarding the results presented in Fig 1A. Specifically, the results presented for iron oxide (F) appear similar to the results presented for glycine capped (GF), glycine and pluronic stabilized (PGF), and cinnamaldehyde tagged (CPGF) when vertically squeezed.

The corresponding author stated that all patterns looked similar upon processing for smothering and for stacking, which indicated that the crystalline structure for Fe_3_O_4_ remains the same after surface modification with the compounds presented in the article. They provided underlying data for editorial review.

The underlying data were assessed by a subject expert who concluded that the underlying data for PGF, GF, and F are identical but scaled by successive factors of ½ and shifted upwards. In addition, the expert commented that the data provided for CPGF look different from the data provided for the other compounds, but that the underlying data provided for CPGF bear no similarity with the pattern labelled as CPGF in the published figure. Furthermore, they commented that if the table labelled as CPGF represents the actual experimental diffraction pattern obtained, then the amount of nanoparticle in the sample is negligibly small.

Following the comments from the subject expert, the corresponding author stated that the XRD data were outsourced to another laboratory and that they were unaware of the issues with the data set. The corresponding author offered to provide replacement data, but the journal does not consider repeat experiment data sufficient to resolve the concerns pertaining to the integrity of the published results.

The data underlying this study remain available upon request from the corresponding author.

In light of the unresolved concerns with Fig 1A that question the reliability and integrity of these data, the *PLOS ONE* Editors retract this article.

All authors did not agree with the retraction.
